# Employment Experiences among Young Malaysian Adults with Learning Disabilities

**DOI:** 10.3390/ijerph17010115

**Published:** 2019-12-23

**Authors:** Dzalani Harun, Normah Che’ Din, Hanif Farhan Mohd Rasdi, Khadijah Shamsuddin

**Affiliations:** 1 Program of Occupational Therapy, Faculty of Health Sciences, Universiti Kebangsaan Malaysia, Jalan Raja Muda Abdul Aziz, Kuala Lumpur 50300, Malaysia; 2 Program of Health Psychology, Faculty of Health Sciences, Universiti Kebangsaan Malaysia, Jalan Raja Muda Abdul Aziz, Kuala Lumpur 50300, Malaysia; 3 Department of Community Health, Faculty of Medicine, Universiti Kebangsaan Malaysia, Jalan Yaacob Latif, Bandar Tun Razak, Kuala Lumpur 56000, Malaysia

**Keywords:** learning disabilities, transition, employment, young adults

## Abstract

The aim of this study was to describe the employment experiences of persons with learning disabilities (LDs) in developing countries, such as Malaysia. Factors associated with respondents’ employment were also determined. A cross-sectional survey was conducted among young adults with LD who left the special education programs in secondary schools in Kuala Lumpur and Selangor. Ninety young adults with LD, aged 18 to 25 years were interviewed face-to-face at an agreed upon convenient place on their working experiences after leaving secondary schools. A total of 13 respondents were excluded from the analysis because their intellectual quotient (IQ) score demonstrated a high possibility of intellectual disability with IQ estimation <70. Of the 77 young adults analyzed, 74.0% reported having work experience and 64.9% were working at the time of interview. Statistical analysis showed significant associations between individual, family, and community factors with respondents’ employment. Two factors made a unique statistically significant contribution to the model (gender, *p* = 0.043 and adult service: Financial support *p* = 0.012). This study suggests the current school-to-work transition program at secondary and post-secondary schools should be improved to better prepare young adults with LD with necessary skills relevant for the current job market so that they could improve their employability.

## 1. Introduction

Participation in employment activities is considered worldwide as one of the important transition outcomes for young adults with disabilities, including those with learning disabilities (LDs). The ability to engage in stable employment is perceived as a successful school-to-work transition outcome [1,2]. Young adults with LD can not only live an independent life but also can contribute to the economic stability of the country [3,4]. Literature on the employment of young adults with LD mainly comes from developed countries, such as the United States and Canada, rather than developing countries, such as Malaysia. 

Many factors are found to be associated with the employment of young adults with LD. These include personal, family, and community factors. The personal factors include age, gender, educational level, and positive attributes. The family factors include parental involvement and expectations, socioeconomic level, and parents’ education while community factors include support services received from the government and/or non-governmental organizations.

Cameto (2005) [5], in reporting employment outcomes of young adults with disabilities two years out of school, found that those who were older were more likely to be engaged in full time employment (*p* < 0.001), were less likely to work in personal-care jobs, and were earning more than USD $7.00 per hour. With regard to gender, female young adults with LD were less likely to engage in employment compared to males [6,7,8]. Moreover, Hogansen et al. (2008) [9] found that parents and educators were concerned about the safety issues of young women with disabilities and indicated the need to restrict the career options and social networks of these young women. As for educational attainment, evidence showed that successful employment of young adults with LD is associated with high academic qualifications, such as bachelor’s degree and/or master’s degree [7,10,11]. Benz et al. (1997) demonstrated that young adults with disabilities (including those with LD) who had high academic skills, reading, writing, or arithmetic skills were two to three times more likely to be employed compared to those with low skill [6].

Individuals’ successful engagement in employment is often influenced by their family characteristics. Whiston and Keller (2004), in their comprehensive review of the literature, found that young adults’ career development was influenced by parental characteristics, such as parents’ education, occupation, and socioeconomic status [12]. Morningstar et al. (1995) suggested that families that include extended family members (i.e., grandparents, aunts, uncles, and siblings) may be considered as influential partners in preparing youths with disabilities for life after school [13]. Rojewski and Kim (2003) found that two-thirds of youth from low socioeconomic status were engaged in employment while two-thirds of those from high socioeconomic status were engaged in post-secondary education [14]. However, Lindstrom et al. (2007) offered a different perspective on how the family socio-economic status influenced young adults with LD [15]. Participants from low socio-economic status in their in-depth case-study investigation were reportedly engaged in early employment. Lindstrom and colleagues revealed that a family low socio-economic status bolstered these participants’ vocational identity and career maturity. The early contribution of these young adults with LD to their family through early employment and care-taking roles may teach them the sense of responsibility and strong work ethic [15].

Parental education has also been found to contribute to their child’s transition outcomes. Parents with low education are found to be less involved in their children’s educational experience [16], which will indirectly influence the child’s transition outcomes, including engagement in employment. Lindstrom et al. (2007) found that parents with low education and did not graduate from high schools demonstrated limited career aspirations for their young adult children with LD [15]. On the other hand, parental education is also found to be associated with their engagement in low-income jobs [17]. Studies showed that young adults whose parents were involved in unskilled and low salary jobs showed limited career interest as compared to those with parents in professional occupations [15,17]. Parents in this study might simply struggle to get enough income for the family and had little time left to focus on the career needs of their young adult with LD [15].

A number of community-related factors can influence young adults with LD transition outcomes. These include the provision of government policy and legislation for transition services, availability of good and effective transition programs, and provision of adult services to support the transition process of persons with LD. A comprehensive transition program for individuals with LD is necessary to ensure a more positive outcome [18,19,20,21]. The transition program should consider important components, such as individualized planning [22,23,24,25], vocational preparation/training [6,26,27], job-seeking and placement [18,25,28], academic remediation and support [7,29,30], counselling [20,31,32,33], support systems and services, and follow-up/follow-along [25,27].

Adult services for young adults with LD should be initiated right after they exit school [28]. It involves the development of contact with other related adult services to ensure the continuity of needed supports for young adults with LD. These services can be very focused, such as vocational training or job training or broader academic or vocational preparation [28]. Parents of young adults with disabilities, including those with LD, reported that their children required vocational assistance, life skill training, tutoring, reading, interpreting, and personal counselling services after leaving secondary/high school [34].

In Malaysia, employment for persons with disabilities is historically seen as a charity act and often stereotyped into welfare cases [3]. Generally, persons with disabilities are marginalized and perceived as less productive. Vocational training centers for young adults with LD are limited [35]. In many instants, work skills-related training is advocated by non-government organizations. Consequently, the development of services and research in this area is very limited. Vocational training for young adults with LD under the Department of Social Welfare (DSW) emphasizes traditional job-related skills, such as handicrafts, carpentry, sewing, cooking, and farming skills [36]. However, in more recent years, the employment needs of persons with disabilities, including those with LD, has become a national focus. This is partly due to the increasing awareness among advocates of this group, including the persons with disabilities themselves, their parents, teachers, services providers, and researchers in this field. Increasing numbers of school leavers from special education programs from government-funded schools may also contribute to this positive change. Many programs have been started to improve the employability of these young adults at secondary schools and post-secondary schools [37,38,39]. The transition unit, which focuses on preparing students for employment, was established at the ministry level. At the secondary school level, the Special Education Division of the Ministry of Education has started collaboration programs with government training institutions, such as community college, People’s Trust Council (MARA) training centers, and private colleges, which aim to equip students with vocational and work-related skills. Students are given opportunities to learn vocational and work-related skills in better facilities and from skillful instructors. At the post-secondary school level, the Department of Social Welfare (DSW) has initiated job placement programs with a few established supermarkets and the fast food industry in the country. The DSW has also initiated the job coach program to improve the job sustainability of young adults with disabilities. The government has established policy and regulations to support the employment of persons with disabilities, including 1% employment policy in the government sectors [40,41]. In addition, the Labor Department of the Ministry of Human Recourse has also started to pay attention to the needs of this group. Persons with disabilities are encouraged to register in the Labor Department’s database for job opportunities: ‘Job placement system for persons with disabilities’ (SPOKU). The department also provides financial assistance for persons with disabilities who are self-employed and plan to expand their business: ‘Business assistance scheme for persons with disabilities’ (SGBP-OKU). 

Although the development of services to support the employment of persons with disabilities is observed to be undertaken seriously by the government and non-government organizations, how those services improve the employment of persons with disabilities is less studied. Generally, persons with disabilities are still perceived by the community as lacking skills and competencies in competitive employment [42]. There is limited research on the employment rates and experiences of persons with disabilities in Malaysia, especially for young adults with LD. This drawback may also contribute to the slow progression in the development of employment-related services, including transition programs at schools and post-secondary schools. Thus, this study aimed to evaluate the employment experiences of young adults with LD who have completed the special education program in the secondary schools system in Malaysia, particularly on their employment outcomes, job stability, and factors associated with the outcome. 

## 2. Materials and Methods

### 2.1. Participants

This study used a quantitative approach using a cross-sectional study design. Participants in this study were school leavers with LD aged 18 to 25 years old who were within the Ministry of Education system, which adopted an inclusive approach to categorize students to be enrolled in the Special Education Program (SEP). These school leavers had been diagnosed by doctors or identified by senior special education teachers to have LD. Selection criteria were set to minimize the heterogeneity of this population in Malaysia by excluding school leavers with autism, cerebral palsy, Down syndrome, intellectual disabilities, and those with a significant sensory disorder. 

### 2.2. Procedure

The young adults with LD who graduated from the special education programs (SEPs) in schools in Kuala Lumpur and Selangor were interviewed face-to-face by the first author either in their home or interview room at the first author’s office on their working experiences after leaving secondary schools. This ‘in-person interview’ [43] is an appropriate method of data collection if the questionnaire is long and when a high response rate is essential. During the interview, the researcher read out the questions of the study questionnaire to the respondents and then filled in the study questionnaire according to the responses/answers given by the respondents. Respondents could ask for clarification on questions that they found not clear or did not understand. This interview approach was considered appropriate as some respondents were having reading difficulty. Approval to conduct this study was sought and received from the Institutional Medical Research and Ethics Committee (FF-270-2010), Ministry of Education Malaysia, State Department of Education, and schools’ management. Thirty schools (15 in Kuala Lumpur and 15 in Selangor State) were involved in this study. The SEP coordinators or senior special teachers of selected schools helped the first author identify potential subjects for the study and provided the researcher with the school leavers with LD parents’ contact number and mailing address. The respondents were contacted by phone call to invite them to take part in this study. For this purpose, one of the researchers, i.e., the first author contacted the respondents’ parent/guardian first, using the contact numbers provided by the schools through the special education teachers. Parents/guardians then gave their verbal consent for their child to participate in this study. Then, the young adults with LD were also contacted for their agreement to participate in the study. Written consent for agreeing to participate in this study was also sought from the young adults prior to administering the questionnaire. For those who were un-contactable through this method, they were invited to participate in this study by invitation letters. Young adults who assented to participate also took the Wechsler Abbreviated Scale of Intelligence (WASI) test, which was conducted to determine their intelligence quotient (IQ) estimation. The test was done by the first author of this study in accordance to the guidelines stated in the WASI manual [44]. The first author, who is a graduate occupational therapist with 15 years working experience, was trained on the WASI standardized testing procedures, including administration procedures, how to score responses, and calculate composite scores (Wechsler, 1999), by the qualified clinical psychologist, who herself is the second author of the paper. The young adults and their parents were informed regarding the procedures involved in the data collection process, i.e., response to the questionnaire and WASI test prior agreeing to participate in the study.

### 2.3. Variables of the Study

The dependent variable of this study was employment. The variable was measured as “Yes”, i.e., engaged in employment, which referred to working in competitive employment/self-employed/family business, either full-time or on part-time basis; and “No”, i.e., not engaged in employment, which referred to working in a supportive/sheltered employment setting or unemployed. For this purpose, participants were asked to respond to the descriptions of the workplace listed in the answer options that were read to them. Competitive employment is described as work in the integrated, competitive setting, where most employees are non-disabled, whereas the supported/sheltered employment setting referred to young adults’ work in the employment setting/environment that provide closed supervision and support services that include work in employment program in the community, day care center, or sheltered workshop. The independent variables were the respondents’ personal characteristics (gender, age, IQ estimation, educational level), their family characteristics (type of family, parent’s monthly income, parents’ education and occupation), and community characteristics (services received during and after secondary schools). The services received during school include type of curriculum taken, type of program attended, work skill training, job seeking training, and academic support. The services received after leaving secondary schools include vocational training, job coach program, independent living training, sheltered employment, pre-employment registration, Business Assistance Scheme by the Labor office department, social skills training, job-seeking and placement, academic remediation and support, counseling, and financial support by the social welfare department. The overall services received referred to young adults with LD usage of services available for them provided by any agencies during and after leaving secondary schools. The variable on services was measured through “Yes”/“No” questions on the types of services received. 

## 3. Results

A total of 90 respondents agreed to participate in this study. Thirteen respondents were excluded from the analysis because their IQ score demonstrated a high possibility of intellectual disability defined as IQ estimation <70. Data from 77 respondents with LD were analyzed using IBM SPSS Statictic 24.

### 3.1. Respondents’ Characteristics and Employment Experience

Of the participants, the majority were Malays (91%) and 9.1% non-Malays (Chinese, Indians, and others). Their age range was between 18 to 25 years (median age 23 and IQR 22–24). The duration of time between school completion and study participation ranged between 2 to 7 years and its median was 4 years. Of these, 61.0% had been out of school for more than 3 years. The majority of respondents did not report having any significant medical illness/condition. Other characteristics are summarized in Table 1. 

**Figure 1:**
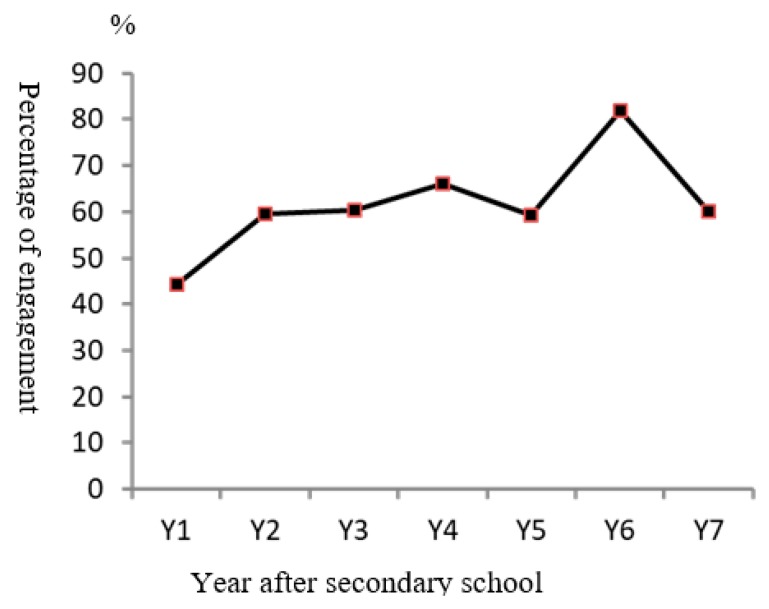
Level of employment engagement by duration of leaving secondary school.

For employment experiences, 74% of respondents had experienced working since leaving secondary school. However, the employment rate of respondents was 64.9% (*n* = 50) at the time of interview. Most of them reported engaging in low paid/elementary job, such as a general worker at shops/petrol stations/supermarkets/companies, dispatch-boys, workers at small industries, and as sale assistants. The characteristics of the respondents’ employment are summarized in Table 2 while the employment stability is shown in Figure 1. It was found that the majority of respondents were engaged in employment in the second year they were out of schools. The number steadily increased but was slightly reduced in the fifth year. An inconsistent pattern of work engagement was observed after the fifth year, with respondents showing a remarkable increased in work engagement at the sixth year (change from 59.3% to 81.8%) and was reduced in the seventh year (change from 81.8% to 60.0%) out of school. 

### 3.2. Factors Associated with Employment

Personal, family, and community factors were found to be associated with respondent engagement in employment. Table 3 shows the significant association of engagement in employment with those factors. Based on the bivariate analysis, seven factors were found significant to engagement in employment. The significant factors are respondents’ gender (personal characteristic), family monthly income, mother’s educational level, and parents’ expectation (family characteristic), financial support, vocational training, and employment-related services and usage of services (community characteristics). 

In total, 73% of male respondents and 68.4% of those aged 21 and younger were engaged in this transition outcome compared to 45.5% of female respondents and 63.8% of those with older age. The differences between male and female respondents’ engagement in this outcome was statistically significant, with a chi square value of x^2^(1) = 5.133, and *p*-value of 0.02. 

Regarding family factors, Table 3 shows 78.1% of respondents with a low family monthly income (≤RM2300) were engaged in employment compared to 55.5% of respondents from middle to high family monthly income (>RM2300). This difference was statistically significant with a Chi-square value of x^2^(1) = 4.184 and *p*-value = 0.04. For the parental factor, 66.1% of respondents with a father with high educational level (secondary and higher education) were engaged in employment compared to 61.1% of respondents with a father with low educational level (primary and lower). Interestingly, the opposite pattern of engagement was reported by respondents with a mother with a low educational level, where 93.3% of them were engaged in employment compared to just 58.2% respondents with a mother with high education; and this difference was found to be statistically significant with a Chi-square value of x^2^(1) = 6.598, and *p*-value = 0.01. For parents’ expectation, 68.6% of respondents whose parents had a moderate to high level of expectation were engaged in employment compared to merely 28.6% of those parents with parents with a low level of expectation. The differences was found to be statistically significant with a Chi square value of x^2^(1) = 4.472 and *p*-value = 0.048 using Fischer’s exact test.

For community factors, interestingly, the chi-square analysis showed that services received at the secondary school level did not have a significant association with the respondents’ post-secondary school employment engagement. This is contrasting to the services received at the post-secondary school level. All respondents who received vocational training and employment-related services were engaged in employment compared to 60.3% who did not receive this service; and this difference was found to be statistically significant with x^2^(1) = 5.503 and *p*-value using Fisher’s exact test of 0.02. Similarly, differences were found between financial support and respondents’ engagement in this outcome, where 90.6% of respondents who received this service were engaged compared to 46.7% who did not receive this service. The difference in this engagement was also found to be statistically significant with x^2^(1) = 15.953 and *p*-value < 0.001. As for respondents’ overall usage of support services, which refers to the number of services received during and after secondary school, 79.3% of respondents from the group with scores above the five types of services received were engaged in employment compared to 56.3% from the group with score five and below. The differences was found to be statistically significant with x^2^(1) = 4.222 and *p*-value = 0.040 (Table 3). 

### 3.3. Regression Analysis

A binary logistic regression was performed to assess the effect of a number of factors on the likelihood that respondents would engage in employment (Table 4). Eight factors, including age, IQ, gender, overall usage of support services, type of family, parents’ monthly income, mother educational level, and adult services; financial support was also included in the model. The full model containing all predictors was statistically significant, x^2^(9) = 33.2, *p* ≤ 0.001, with a Nagelkerke R square of 48.3%. However, only two factors made a unique statistically significant contribution to the model (gender, *p* = 0.043 and adult service: financial support *p* = 0.012). The model showed that male respondents, compared to female, were 11 times more likely to engage and respondents who received adult service and financial support, compared to those who did not receive, were 5 times more likely to engage in employment. 

## 4. Discussion

The discussion on findings of this study is delimitated to how employment contributes to the economics of the country. It is suggested for others to look into the relationship and contribution of young adults’ engagement in work towards their self, families, community organizations, government, and educational system of the country.

The employment rate of persons with disability in Malaysia is commonly reported as low [3,42]. Findings from this study showed that 64.9% of respondents were engaged in employment (full-time and part-time) at the time of survey. Seventy-four percent had experienced working since leaving secondary school and the majority of them worked in competitive employment. While this number appeared encouraging and comparable to findings from overseas [45,46], the fact is that this rate is perceived as disappointing when compared to their peers without disabilities [47]. With the exception of studies by Madaus [7,48], most overseas studies reported unsatisfactory employment outcomes of school leavers with LD. Moreover, the employment quality of respondents’ in this study, which included type of job, salary, job benefits, and job satisfaction, which are equally important, was also disappointing. The majority of respondents engaged in low-earning unskilled jobs (security guard, general workers in retail and service industries), and received a monthly salary below the national minimum wage, and 8% received a salary of RM500 (USD167) and below. As a result, the majority of respondents in this study are at risk for dependent living. Although the government provided financial support for these young adults through an allowance for workers with disabilities, what is equally important is to provide is a means to increase their employability in the competitive job market. It is critical for persons with disabilities to equip themselves with criteria for employment that include basic and advanced academic qualification, living and vocational skills, and motivation to work [6,18,27]. It is also important for persons with disabilities to learn how to find job that is appropriate with their aptitude, skills, and interest to ensure getting suitable, well-paid jobs. Moreover, not all the respondents receive the job allowance given by SWD. Some were not aware of this job incentive and some were troubled with the bureaucracy in getting this incentive.

A lack of job-seeking skills is another challenge that may be experienced by the young adults in this study. The majority of them got their current jobs through someone’s assistance or they were recommended by their parents/family/friends or family’s/friend’s network, rehabilitation agency, and school/teacher. This pattern of depending on personal/family-friend network, employment agency, and teacher/school to find jobs was reported in previous studies [46,49]. In addition, the respondents’ low academic qualifications and low job skills may have also contributed to this pattern. Abdul Rahman Embong (2011) found that in an urban population, such as in the Kuala Lumpur city, those persons having low academic achievements (junior and senior secondary qualification, PMR and SPM) are mainly engaged in manual work and got their job through recommendations from family members/friends [50]. Moreover, the negative perception of employers on persons with LD is also another possible contributor to this state of situation [38,42]. The Malaysian society needs to change their stereotyped perception of persons with disabilities on welfare cases. They should be fully accepted as part of the country’s employment force and given the equal opportunity to contribute to the country’s economy.

In addition, a significant proportion of the respondents in this study wanted to work but reported that they did not have the prerequisite for work and opportunities to do so. This scenario needs to be intervened so that it does not contribute to a loss of income for the country. Unemployment would also result in these young adults with LD continuing to depend on their parents/family or government and jeopardize their transition goals for independent living. As highlighted by Khor (2002), the exclusion of persons with disabilities from mainstream society as estimated by the World Bank to result in a total loss country income between US$1370 billion and US$1940 billion. For Malaysia, the rate is estimated to be between US$1.18 billion and US$1.68 billion. Thus, not only should wider employment opportunities be made available [51,52], increasing employability as well as job advancement for persons with disabilities, including those with LD, is critical in this country.

The discussion on the employment characteristics of the respondents is incomplete without pointing out its limitation. The study did not include a control group of typical young adults and is therefore unable to show the magnitude of the employment problem by a comparison with young adults without disabilities. Statistics on employment in Malaysia are often reported using a wider young adult age range, 18 to 40 years old, which make the comparison misleading. Thus, it is highly recommended that a future study further investigates this topic.

As for factors associated with employment, this study revealed that respondents’ family and community characteristics are equally significantly associated with respondents’ engagement in employment compared to individual characteristics. For individual factors, gender (male) was significantly associated with respondents’ engagement in employment (*p* = 0.02). This finding is consistent with many other studies on employment among persons with LD globally [6,7,8,45,46]. The significant association can be understood as more males participated in this study compared to females. Moreover, a bigger proportion of male respondents were employed compared to female respondents at the time of interview. It is interesting to note that other individual factors, such as age, and educational level, that were hypothesized to be associated with employment failed to show their significant association with this outcome. This could be due to differences in the various aspects of this study compared to the previous studies that showed significant associations of those factors with employment. For educational level, the respondents’ education level in this study was mainly primary and secondary academic qualification. This is dissimilar when compared to other studies that showed significant outcomes, such as respondents who completed high school in Wagner et al. (2005) [45], respondents with bachelor’s or master’s degrees compared to high school diplomas in Goldberg et al. (2003) [10], and high academic skills, including reading, writing, or arithmetic skills, in Benz et al. (1997) [6]. The big differences between respondents’ educational level/academic attainment in this study and the other mentioned studies may contribute to the non-significant finding between the respondents’ education level and employment in this study.

With regard to family characteristics, the current study shows that the mothers’ educational level instead of the fathers has a greater influence on the respondents’ engagement in the transition outcomes. More interestingly, the chi square analysis shows that respondents with mothers with low educational levels were significantly more engaged in employment (*p* < 0.01). This finding is contrary to the findings in the previous studies by Levine and Wagner (2005), who found that high parental education level, especially of the head of the family, is associated with children having better employment outcomes [53]. This interesting finding may indicate that mothers in this study had a closer relationship with their children and had more influence on their children’s employment. Since the majority of the respondents’ mothers were full-time housewives, they may have more time to encourage and support their children to work.

Moreover, respondents in this study may have been motivated to improve their socio-economic status or standard as the majority of them come from family with a low socio-economic background. They may have experienced a hard life having grown up in a large family with financial instability, especially when the fathers were the sole income earners for the family. They may also learn that they could no longer rely on financial support from their parents. The positive characteristic may be similar to what Lindstrom et al. (2007) suggested of participants in her study, who had grown up in families with low socio economic backgrounds and yet were successfully engaged in employment [15]. Similar to participants in Lindstrom et al.’s (2007) study, respondents in this study wanted to “be different” and were motivated to have a better life, to engage in steady jobs and have stable life outcomes [15]. In addition, it is also probable that the respondents were expected to help with the family financial needs and family functioning (Lindstrom et al., 2007) [15]. It was found during the interviews that many of the male respondents were helping their parents with the family’s financial needs and tried not to burden their parents with their own personal needs. These findings may help us understand the dynamic relationship between respondents’ individual and family factors that may directly or indirectly be associated with their engagement in employment. However, more studies on this subject need to be undertaken before an association between the young adults with LD individual’s motivation and their parental characteristics is more clearly understood in east populations, like Malaysian. Transition planning is known to be an important element for a successful transition outcome, including employment. Thus, it is suggested that transition planning for young adults with learning disabilities should actively involve their parents regardless of their parents’ education level.

For community factors, ‘vocational training and employment related services’, ‘financial support’, and ‘usage of services’ (*p* = 0.04) were found to be statistically associated with respondents’ employment. The finding on the association of receiving vocational training and employment-related services with respondents’ employment is aligned with the finding from overseas studies [6,27]. Specifically, this study showed that respondents who received vocational training, job coaching project, vocational/career counselling, and placement system for people with disabilities) were significantly more engaged in employment (*p* = 0.02) although the numbers were small. Thus, this finding has important implications for developing post-secondary school training for young adults with LD in the country. Echoing what was suggested by Loh and Sharifah Zainiyah (2013) [35], this study strongly suggests that more vocational training programs should be provided for young adults with LD in the country at the post-secondary school level.

In Malaysia, financial support is a well-known type of service provided by the SWD for persons with disabilities. This includes a RM400 (USD105) monthly allowance for workers with disabilities who earn less than RM1200 (USD400) a month. As a result, the majority of employed respondents in this study had received this support service. Thus, it is of no surprise that this finding shows that respondents’ engagement in employment was significantly associated with this variable (*p* = 0.001). The result indicates that an incentive in the form of the financial support that is currently provided by the SWD is successful in encouraging young adults with LD to engage in employment and thus contribute to the country’s economic development.

The determinants for engagement in employment in this study are ‘male’ and ‘receiving financial support’. Male as a predictor for employment outcome for persons with LD is not a surprise as globally, males are more prevalent in LD. In addition, the workforce in Malaysia in general is dominated by male workers especially for elementary/manual labor work, and culturally, adult males in the family, especially the eldest, are expected to contribute to the family’s financial needs. This trend is also similar among persons with disabilities [54]. Nevertheless, the majority of female respondents in this study wanted to work and experience life just like their male counterparts and peers in the general population.

As for receiving financial support, this finding suggests SWD should not only maintain this service but further improve this service by increasing the monthly threshold salary limit, from less than RM1200 (USD400) to RM1500 (USD500), taking into consideration the increasing cost of living and to further minimize the bureaucracy involved in its implementation. The amount of the allowance also needs to be revised to make it more relevant to the current economic changes.

## 5. Recommendations

Findings from this study support the recommendation to provide vocational rehabilitation programs for young adults with LD who require more comprehensive training courses [1,2,28,47]. The training program should incorporate other necessary skills, such as social skills training, living skills training, and survival skills training, and follow up services that were found to be important for successful employment. It is also suggested that more opportunities should be given to school leavers with LD to enroll in the current training programs by government training agencies and institutions under the Ministry of Human Resource, Ministry of Education, and MARA, as well as private educational and training institutions. For those who need more inclusive training, it is suggested that SWD provides vocational rehabilitation training programs that include vocational evaluation and training by trained and qualified professionals, such as occupational therapists and vocational counsellors [55].

With regard to the small number of female young adults with LD involved in the country workforce, this study recommends for future studies to further investigate this area and determine factors that contribute to this low employment rate. It is also proposes that rehabilitation and training programs are in line with the current country’ industries demands. This is to ensure that young adults with LD are equipped with the necessary skills (work/vocational skills, good work habit, social and survival skills) for successful employment [6,22]. It is also recommended that vocational training includes courses that address the occupational needs of females with LD. Courses, such as child/nursery care and elderly care, can be taught so that they have skills to work as assistant teachers, and staff or assistant staff at nursing homes or nurseries. A systematic job coach program/follow-up service is also another component in vocational rehabilitation programs that should be made widely available to ensure that these young adults can successfully manage difficulties at work and daily living and sustain their employment.

## 6. Conclusions

As a whole, this paper increased our understanding on the employment experience of Malaysian young adults with LD; the characteristics, challenges, and factors that associated with their employment. It clearly demonstrates that the current practice of vocational training for persons with LD in Malaysia needs to be further improved. Training programs should consider more up-to date vocational/employment skills, such as computer and information communication technology knowledge and skills, which will better prepare them for employment in the current market demands. Recommendations for more comprehensive post-secondary school vocational and work skill training can be considered by the involved agencies, government or non-government, to ensure that Malaysian young adults with LD are better prepared for sustainable employment.

## Figures and Tables

**Table 1:** Socio-demography characteristics of respondents (*n* = 77).

Characteristics	*n*	%
Gender		
Male	55	71.4
Female	22	28.6
Age (years)		
21 or younger	51	66.2
more than 21–25	26	33.8
Min–Max	18–25	
Median, IQR	23.0 (22.0–24.0)	
WASI 1Q score		
70–84	34	44.2
85–90	22	28.6
91 and above	21	27.3
Min–Max		70–104
Median, IQR	85 (74.0–92.0)	
Educational level		
Certificate of ending school	16	20.8
PMR	42	54.5
SKM/SPM	19	24.7
Year of leaving school(years)		
3 or less	30	39.0
4–7	47	61.0
Min–Max	2–7	
Median, IQR	4 (2.0–5.0)	
Presence of co-morbidity		
Yes	14	18.2
No	63	81.8
Current living arrangement		
Alone in rented house/apartment/room	3	3.9
With spouse or roommate in a home/apartment	9	11.7
With parent/guardian	60	77.9
With other family members	4	5.2
College or work hostel/accommodation	1	1.3

**Table 2:** Characteristics of respondents (*n* = 77).

Characteristics	*F*	%
**Ever employed since leaving school**	**(*n* = 77)**	
Yes	57	74.0
No	20	26.0
**Current employment status**		
Employed	50	64.9
Unemployed	27	35.1
**No. currently employed**	**(*n* = 50)**	
	***F***	**%**
**Way of getting current job**		
Self	12	24.0
School/teacher	2	4.0
Rehabilitation Agency	7	14.0
Family, family/friend network	29	58.0
**Type of current job**		
Competitive	46	92.0
Supported/from home	1	2.0
Other (family business)	3	6.0
**Nature of current job**		
Full time	45	90.0
Part time	5	10.0
**Working shift**		
Yes	21	42.0
No	29	58.0
**Duration of current job (month)**		
3 and less	10	20.0
More than 3–11	13	26.0
12 and longer	27	54.0
**Working hours per day (hours)**		
8 and less	34	68.0
More than 8-9	12	24.0
10 and more	4	8.0
Min–Max	4–14/8 (8.0–9.0)	
Median, IQR		
**Working days per week(days)**		
5	11	22.0
6	37	74.0
7	2	4.0
Min–max/Median, IQR	5–7/6 (6–6)	
**Salary per-month** (RM)		
500 or less	4	8.0
More than 500–1000	33	66.0
More than 1000 -1500	8	16.0
1501 and more	5	10.0
Min–Max	RM150.00–1800.00	
Median, IQR	RM875.00 (700.00–1050.00)	
Work benefits received *		
*SOCSO/health insurance*	41	82.0
*EPF/pension*	41	82.0
*Annual leave*	35	70.0
*Medical leave*	38	76.0
*Allowance*	20	40.0
*Bonus*	27	54.0
*Others (food, accommodation)*	7	14.0

* *SOCSO* = Social security insurance, *EPF* = Employee Provident Fund.

**Table 3:** Significant differences in the prevalence of engagement in employment (n = 77).

Characteristics	Employment			
	*n*	Employed *N* (%)	Chi square	*p* Values
**I. INDIVIDUAL**				
**Gender:**				
Male	55	40 (72.7)	x^2^ = 5.1333	* *p* = 0.02
Female	22	10 (45.5)		
**II. FAMILY**				
**Family monthly income:**				
Low (≤RM2300)	32	25 (78.1)	x^2^ = 4.184	* *p* = 0.04
Middle to high (>RM2300)	45	25 (55.6)		
**Mother educational level:**				
Low (Primary and lower)	15	14 (93.3)	x^2^ = 6.598	*p = 0.01
High (Secondary and higher)	62	36 (58.1)		
Vocational training and employment related services **				
Yes	9	9 (100.0)	x^2^ = 5.503	*^,^*^a^ p* = 0.02
No	68	41 (60.3)		
**Parent expectation**				
Low	7	2 (28.6)	x^2^ = 4.472	*^,^*^a^ p* = 0.048
Moderate to High	70	48 (68.6)		
Financial support ***				
Yes	32	29 (90.6)	x^2^ = 15.953	* *p <* 0.001
No	45	21 (46.7)		
**Overall usage of support services:**				
Low (≤5 types of service)	48	27 (56.3)	x^2^ = 4.222	* *p* = 0.04
High (>5 types of service)	29	23 (79.3)		

*^a^ p* value using Fisher’s Exact Test; * values are significant at *p*-value < 0.05. For analyses purposes: ** Vocational training & employment related service was included Vocational training, Job coaching project, vocational/career counselling, and placement system for person with disabilities. *** Financial support service was included allowance for college students/workers with disabilities and Business assistance scheme for person with disabilities.

**Table 4:** Factors for predicting employment.

Factors	OR	95% CI	
		Lower	Upper
**Individual**			
*Male* vs. *Female*	4.90	1.12	99.13
**Community**			
Financial support			
*Received* vs. *Not received*	10.83	1.70	69.16
Constant = 0.028			
Cox & Snell R^2^ = 0.351			
Nagelkerke R^2^ = 0.483			
Model x^2^(df)/*p*-value = 33.26(9)/*p* < 0.01			
H & L test *p*-value = 0.14			
